# Can traditional oral hygiene methods compete with conventional toothbrushes in effectiveness?

**DOI:** 10.1038/s41432-024-01030-6

**Published:** 2024-06-28

**Authors:** Waleed Almutairi, Brett Duane

**Affiliations:** 1 Trinity College Dublin, Dublin, Ireland; 2 Dublin Dental University Hospital, Trinity College Dublin, Dublin, Republic of Ireland

**Keywords:** Periodontics, Dental public health

## Abstract

**Design:**

The study was designed as a single-blinded, parallel, randomized controlled trial to compare the effectiveness of the Salvadora persica toothbrush (MTB), Salvadora persica chewing stick (MCS), and a standard toothbrush (STB) in controlling plaque and gingivitis. A total of 78 participants were randomly divided into three groups and instructed to use their assigned oral hygiene tool in a standardized manner for three.

**Case selection:**

Participants were non-dental students and staff of Universiti Kebangsaan Malaysia, Kuala Lumpur Campus, selected through convenience sampling. They met specific inclusion criteria, such as being systemically healthy, having ≥20 teeth, and having a Basic Periodontal Examination score of 0, 1, or 2, with no periodontal pockets greater than 5.5 mm.

**Data analysis:**

Clinical outcomes were measured using the Plaque Index (PI) and Periodontal Inflamed Surface Area (PISA) at baseline, one-, and three-weeks post-intervention. Data analysis was performed using mixed-model analysis of variance for continuous variables and Fisher’s exact test for categorical variables.

**Results:**

All three groups showed significant improvements in plaque levels and severity of gingivitis from baseline to three weeks post-intervention. The MCS group demonstrated a significant improvement in mean PISA values of the anterior teeth compared to the MTB and STB groups. However, there was no significant difference in plaque level reduction or overall gingivitis severity among the three groups. This indicates that when used correctly, Salvadora persica toothbrushes and chewing sticks are as effective as standard toothbrushes in plaque control and gingival health.

**Conclusions:**

The study concludes that both Salvadora persica toothbrushes and chewing sticks can serve as effective alternatives to the standard toothbrush for plaque control and gingival health. This showcases the beneficial anti-plaque and anti-gingivitis properties of Salvadora persica. However, the effectiveness of these oral hygiene tools is contingent upon the correct usage techniques.

## A Commentary on

**Azizan N F, Mohd N, Nik Azis N M, Baharin B**.

Effectiveness of Salvadora persica toothbrush and Salvadora persica chewing stick in plaque and gingivitis control: a randomized control trial. *BMC Complement Med Ther* 2023; **23:** 456.

**GRADE rating:** 

## Commentary

Dental biofilm, which consists of a complex microbial community, is known to play a significant role in developing dental conditions such as caries, gingivitis, and periodontitis^1^. Therefore, preventive measures involve disrupting dental plaque biofilms and reducing bacterial load through effective mechanical oral hygiene practices like toothbrushing were used^2^. Salvadora persica chewing sticks are natural and affordable, and therefore, preventive measures involve disrupting dental plaque biofilms and reducing bacterial load through effective mechanical oral hygiene practices such as toothbrushing^3^. Cultural and religious traditions often influence the choice of oral hygiene tools, like Salvadora persica, which has a long history of use^3^. Recognizing its efficacy, the World Health Organization endorses Salvadora persica as a valuable oral hygiene tool, particularly in regions where it is customary^4^. Studies have shown the significant role of Salvadora persica chewing sticks in promoting oral hygiene, particularly in areas facing financial constraints or limited oral healthcare resources^5^.

This RCT conducted by Azizan NF et al. provides valuable insights into the efficacy of traditional oral hygiene methods compared to conventional toothbrushes^6^. The study rigorously evaluates Salvadora persica toothbrush (MTB) and chewing stick (MCS) against a standard toothbrush (STB) in a specific population at University Kebangsaan Malaysia^6^. The methodology demonstrates a strong design through its randomization process and intention-to-treat analysis, ensuring reliability and reducing systematic bias^6^. The findings suggest that Salvadora persica products can be as effective as standard toothbrushes in controlling plaque and gingivitis, providing a viable alternative for individuals seeking natural oral hygiene options^6^. This could significantly impact dental practices, particularly in promoting sustainable and environmentally friendly oral care solutions. However, the limitations in participant blinding and the study’s generalizability due to a specific demographic focus introduce areas for caution. These factors and the absence of detailed precision in effect estimates highlight the need to interpret the results carefully.

The potential reduction in fluoride exposure with the transition from standard toothbrushes underscores the necessity of a balanced approach to oral hygiene recommendations. Moreover, this RCT focused on the short-term effectiveness of Salvadora persica products, therefore, Furthermore, this RCT examined the short-term effectiveness of Salvadora persica products. Therefore, conducting additional research on the long-term use of these natural oral hygiene methods would offer a better understanding of their sustained effectiveness and safety. The study contributes valuable insights into the effectiveness of traditional oral hygiene methods, highlighting their potential role alongside conventional practices. Despite its methodological adaptability and significant findings, the study’s scope for wider application necessitates further exploration. As the dental community continues to seek sustainable and effective oral hygiene solutions, this research marks an important step towards understanding the potential of natural products in oral health care.

Practice points

- Prioritize educating patients on the correct techniques for using Salvadora persica products, enhancing their effectiveness with comprehensive instructions and demonstrations.
- Adopt Salvadora persica oral hygiene products to lessen plastic waste, supporting eco-friendly practices that align with sustainability goals.
